# Light-Induced Changes in RGB Reflectance Parameters in Wheat and Pea Leaves in the Minute Range

**DOI:** 10.3390/plants15081184

**Published:** 2026-04-12

**Authors:** Yuriy Zolin, Alyona Popova, Lyubov Yudina, Leonid Andryushaev, Vladimir Sukhov, Ekaterina Sukhova

**Affiliations:** Department of Biophysics, N.I. Lobachevsky State University of Nizhny Novgorod, 603950 Nizhny Novgorod, Russia; uchebnayap.zolin@gmail.com (Y.Z.); silverkumiho@mail.ru (A.P.); lyubovsurova@mail.ru (L.Y.); andoverleanid@yandex.ru (L.A.); vssuh@mail.ru (V.S.)

**Keywords:** leaf RGB reflectance, visible light, photosynthetic light reactions, wheat, pea, optical leaf model, photosynthetic model, RGB imaging, proximal sensing

## Abstract

Parameters of reflected light, measured in narrow or broad spectral bands, are widely analyzed for remote and proximal sensing of plant responses to stressors. Specifically, parameters of reflectance in red (R), green (G), and blue (B) spectral bands measured using simple color images can be sensitive to characteristics of plants. The conventional view is that RGB reflectance primarily reveals long-term changes in plants (days, weeks, etc.). In this study, we investigated light-induced changes in RGB reflectance in wheat (*Triticum aestivum* L.) and pea (*Pisum sativum* L.) leaves. Illumination increased this reflectance for about 10 min in wheat and about 15–20 min in pea; these changes relaxed after light intensity was decreased. The changes in RGB reflectance were strongly related to the effective quantum yield of photosystem II and non-photochemical quenching of chlorophyll fluorescence under high light intensity; these relations were absent under low light intensity. We hypothesized that changes in both RGB reflectance and photosynthetic parameters were related to the light-induced changes in chloroplast localization. A simple mathematical model of optical properties and photosynthesis in leaves was developed; results of the model-based analysis supported the proposed hypothesis. Experimental analysis of the dynamics of light transmittance additionally supported this hypothesis. Our results thus show that RGB imaging can be sensitive to fast changes in plants.

## 1. Introduction

Light influences plant productivity, growth, development, adaptation to environmental conditions, and many other important processes [1,2,3,4]. Specifically, plants transform light into chemical bond energy via photosynthesis [1,5]; the spectrum and intensity of light can regulate processes of differentiation [4,6,7] and induce stress responses [2]. Given the importance of light, plant leaf structure is optimized to use this energy [2,3].

It is known that the plant leaf is a complex medium for light propagation. Light can be absorbed by pigments and water [8]. Leaf absorption properties can be changed through physiological processes, including a decrease/increase in pigment content [9,10], shifts in chloroplast localization [11,12,13], chloroplast shrinkage [14,15], water content changes [8,16,17], and others. Additionally, the complex structure of the leaf, which includes water and air spaces and microstructures in cells, causes multiple scattering [18,19], which modifies light absorption and changes the spectra of reflected and transmitted light. Finally, the roughness of the leaf surface is related to its light transmission and reflectance [20] and, therefore, can also influence light absorption in leaves.

Many noted structural properties and processes can be affected by environmental factors (especially the action of stressors) [9]. This action can induce simultaneous changes in light absorption, reflectance, and transmittance [2,3], providing the basis of development of optical methods of plant proximal and remote sensing [9,21]. Specifically, spectral parameters of reflected light are widely used to detect the influence of abiotic stressors [16] and plant diseases [22]. Visible light is especially interesting for plant proximal and remote sensing because it can be measured using cameras and sensors, which are more available than those used for measurements of reflectance in the infrared spectral region [17]. They can be localized on different platforms, including handheld devices, phenotyping systems, unmanned aerial vehicles, planes, satellites, and others [17,21].

Measurements of reflectance at different specific wavelengths (in narrow spectral bands) can show changes in activity of corresponding processes and can be used to analyze details of stress responses, including shifts in contents of photosynthetic pigments and ratios of these contents, xanthophyll cycle activation, anthocyanin production, etc. [16,17]. However, reflectance in expanded spectral bands can also be sensitive to stress changes in plants; specifically, this sensitivity is observed when measuring broad spectral ranges (up to 100 nm) [23,24]. It means that reflectance in the broad spectral bands can also be used to detect changes in plants [16,25,26,27]; this reflectance approximately corresponds to reflectance in red (R), green (G), and blue (B) spectral bands.

As a result, methods of RGB imaging, which only require simple and low-cost color cameras for measurements, are dynamically developed [28]. RGB imaging is used for sensing of slowly changing characteristics of plants, including chlorophyll concentration [29], nitrogen content [30], above-ground plant biomass [31,32] and others. Specifically, changes in parameters of RGB reflectance are related to plant productivity, their development and maturation (including accumulation of organic substances), and long-term stress changes; at that, the periodic measurements often continue for many days, weeks, and months [31,33,34].

However, the efficiency of using RGB imaging for sensing of rapidly forming and short-term changes has been weakly investigated to date. We earlier showed that electrical signals induce changes in reflectance in broad spectral bands (100 nm), which are analogs of R, G, and B spectral bands; the effect is formed for 30 min after induction of electrical signals and relaxes after that [35,36]. These changes can be related to small shifts in water content in leaves caused by electrical signals [36]; however, other mechanisms of broadband reflectance changes cannot be fully excluded.

Additionally, there are theoretical points supporting the possibility of relatively fast changes in reflectance in R, G, and B spectral bands (for several minutes or tens of minutes). It is known that light-induced changes in chloroplast localization can decrease light absorption in the leaf for minutes [37,38]. These localization changes and decrease in light absorption in the leaf are expected to influence RGB reflectance, i.e., the influence of light can be potentially detected via RGB imaging. Moreover, changes in chloroplast localization are related to photosynthetic light reactions [39,40,41], meaning that RGB imaging is also likely to be sensitive to photosynthetic parameters; however, this point is also weakly investigated.

Our current investigation thus aimed to study the fast influence (range: minutes and tens of minutes) of light intensity on parameters of reflectance in R, G, and B spectral bands in wheat and pea leaves and analyze how these changes related to light-induced changes in parameters of photosynthetic light reactions.

## 2. Results

### 2.1. Light-Induced Changes in RGB Reflectance

In the first stage of the investigation, initial reflectance in R, G, and B spectral bands, which were normalized using the gray reflectance standard (see Section 4.2), was measured in wheat and pea plants for all measured values (*n* = 144 for wheat and *n* = 56 for pea). These average values were 0.745 ± 0.006, 1.200 ± 0.006, and 0.566 ± 0.004 for R, G, and B, respectively, in wheat plants and 0.459 ± 0.011, 0.945 ± 0.017, and 0.313 ± 0.005 for R, G, and B, respectively, in pea plants; i.e., initial reflectance in R, G, and B spectral bands in wheat was significantly higher than this reflectance in pea (approximately on 62, 27, and 81%). Using the ratio between reflectance of gray and white standards (about 0.23, see Section 4.2), we calculated absolute values of initial RGB reflectance: about 0.171, 0.276, and 0.130 for R, G, and B, respectively, in wheat plants and about 0.106, 0.217, and 0.072 for R, G, and B, respectively, in pea plants.

**Figure 1:**
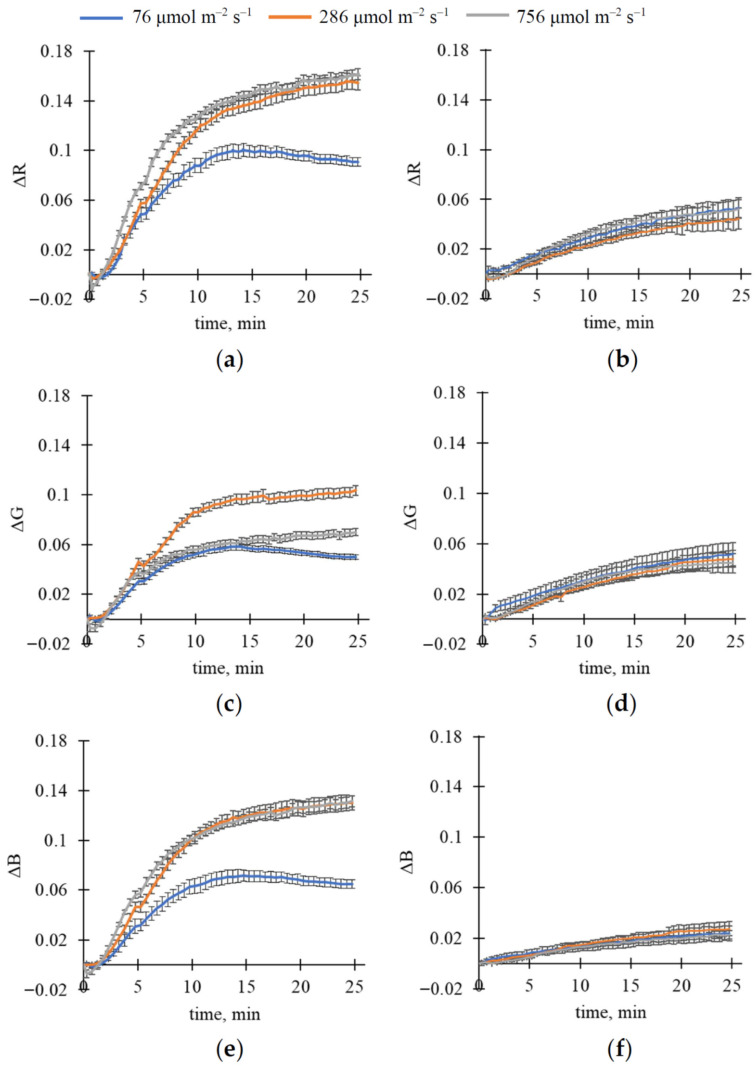
The influence of the 25 min illumination with constant light intensity (76, 286, and 756 μmol m^−2^ s^−1^) on reflectance in red (R), green (G), and blue (B) spectral bands normalized using the gray reflectance standard. Average changes in red reflectance (ΔR) in wheat (**a**) and pea (**b**), in green reflectance (ΔG) in wheat (**c**) and pea (**d**), and in blue reflectance (ΔB) in wheat (**e**) and pea (**f**) are shown. A total of 12 wheat and 6 pea plants were analyzed (*n* = 12 and *n* = 6). ΔR, ΔG, and ΔB were calculated as differences between values measured in the current time and those measured immediately after turning on the light. Average values and standard errors are shown in the figure.

After that, we investigated changes in reflectance in R, G, and B spectral bands (ΔR, ΔG, and ΔB, respectively) induced by illumination with constant intensity (Figure 1); values were normalized using the gray reflectance standard. The changes were calculated as differences between values measured in the current time and those measured immediately after turning on the light.

It was shown that illumination caused increases in ΔR (Figure 1a), ΔG (Figure 1c), and ΔB (Figure 1e) in wheat leaves; we observed a plateau in the increase after approximately 10 min of illumination. Magnitudes of increasing ΔR, ΔG, and ΔB in pea leaves were smaller than those in wheat leaves (Figure 1b,d,f). The dynamics were slow; saturation was unstable and observed approximately 15–20 min after illumination in different cases.

**Figure 2:**
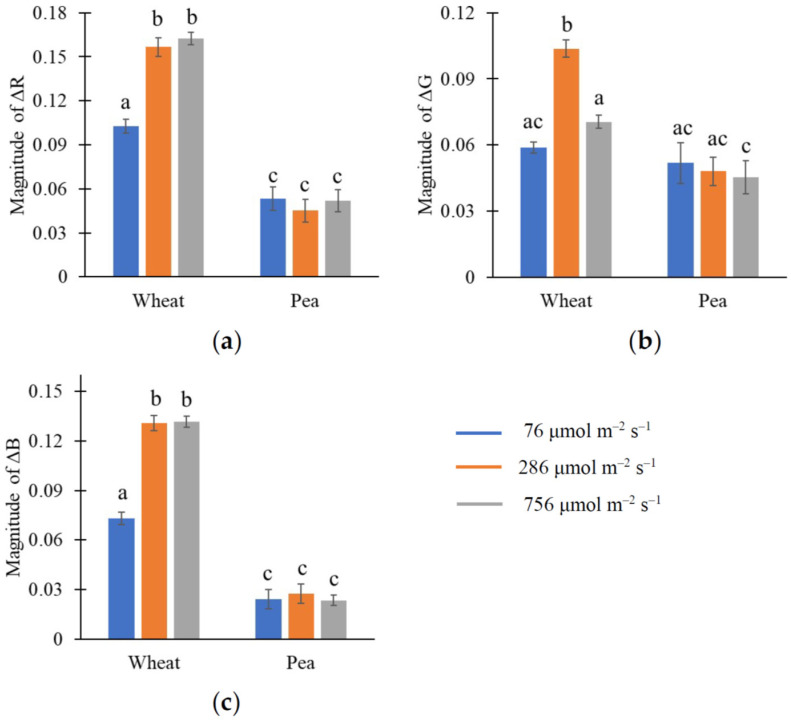
The magnitudes of ΔR (**a**), ΔG (**b**), and ΔB (**c**) under the different light intensities in wheat and pea. A total of 12 wheat and 6 pea plants were analyzed (*n* = 12 and *n* = 6). Magnitudes were calculated as the maximal ΔR, ΔG, and ΔB. The letters show significance between groups (Tukey’s honestly significant difference (HSD) test). Average values and standard errors are shown in the figure.

Figure 1 shows that plant species (wheat or pea) and, probably, light intensity (76, 286, or 756 μmol m^−2^ s^−1^) could influence light-induced changes in reflectance in R, G, and B spectral bands. Analyzing magnitudes of ΔR, ΔG, and ΔB (calculated as maximal ΔR, ΔG, and ΔB) using two-way ANOVA (Table 1) showed that both light intensity and plant species significantly influenced them; moreover, interaction between these factors was also shown. Further analysis of these magnitudes using Tukey’s honestly significant difference (HSD) test (Figure 2) showed that the magnitudes of ΔR, ΔG, and ΔB differed significantly in wheat and pea leaves. Increasing light intensity increased the magnitudes of these light-induced changes in reflectance in wheat plants; however, this effect was absent in pea plants.

Relative magnitudes of increase in reflectance (compared to initial values of this reflectance) were approximately 14–21%, 5–8%, and 14–23% in the R, G, and B spectral bands in wheat plants and approximately 9–13%, 4.5–6%, and 7.5–8.5% in the R, G, and B spectral bands in pea plants, respectively (Table 2).

Further, we analyzed the following question: Could ΔR, ΔG, and ΔB be relaxed after decreasing light intensity? To answer this, we used a light regime comprising three steps with different light intensities: 76 μmol m^−2^ s^−1^ light intensity (20 or 10 min), 756 μmol m^−2^ s^−1^ light intensity (20 min), 76 μmol m^−2^ s^−1^ light intensity (10 or 20 min). The total measurement duration was 50 min. The final ΔR, ΔG, and ΔB values at the first step of illumination (with low light intensity) were used to estimate the basic levels of these parameters. The second step of illumination (with high light intensity) should induce additional changes in RGB reflectance. The third step of illumination (with low light intensity) was used to induce relaxation of additional ΔR, ΔG, and ΔB.

Initially, we calculated ΔR, ΔG, and ΔB using values of reflectance in R, G, and B spectral bands—which were measured immediately after turning on the light (the first step)—as the zero values. It was shown (Figures S1 and S2) that the dynamics of RGB reflectance included light-induced changes formed for minutes or tens of minutes, which corresponded to ΔR, ΔG, and ΔB, as described above.

However, ultra-fast light-induced changes (<several seconds), which could not be compared to changes in RGB reflectance in minute ranges (see above), were also shown (Figures S1 and S2). Dynamics of these ultra-fast changes could not be measured using the current experimental procedure. We only observed differences between parameters of RGB reflectance measured under the current light intensity and those measured under the previous light intensity; varying RGB camera exposure times were used for these intensities. As a result, we could not confidently determine whether the ultra-fast changes were plant responses or artifacts caused by changes in exposure time, meaning that these changes require special investigation in the future; therefore, they were excluded from the current analysis.

**Figure 3:**
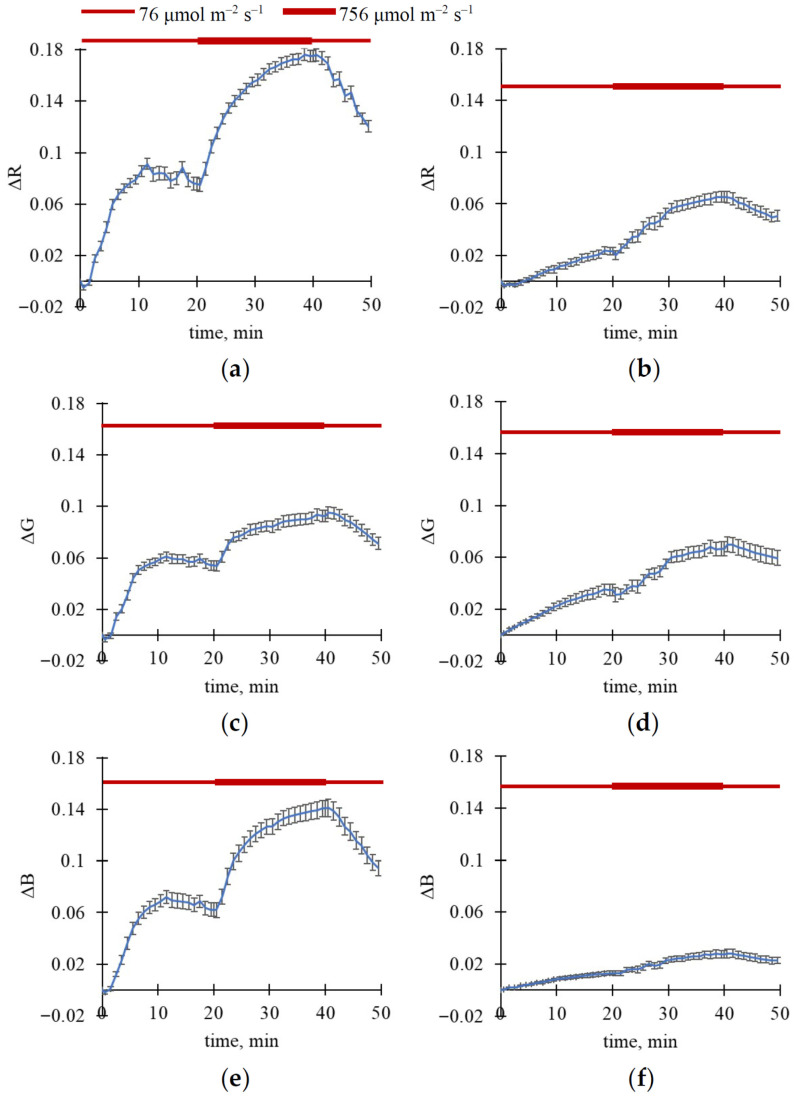
Dynamics of average ΔR, ΔG, and ΔB under three light intensity steps (20 min for 76 μmol m^−2^ s^−1^, 20 min for 756 μmol m^−2^ s^−1^, and 10 min for 76 μmol m^−2^ s^−1^). ΔR in wheat (**a**) and pea (**b**), ΔG in wheat (**c**) and pea (**d**), and ΔB in wheat (**e**) and pea (**f**) are shown. A total of 11 wheat and 4 pea plants were analyzed (*n* = 11 and *n* = 4). ΔR, ΔG, and ΔB were calculated for each light step as differences between values measured in the current time and those measured immediately after step initiation. Average values and standard errors are shown in the figure.

**Figure 4:**
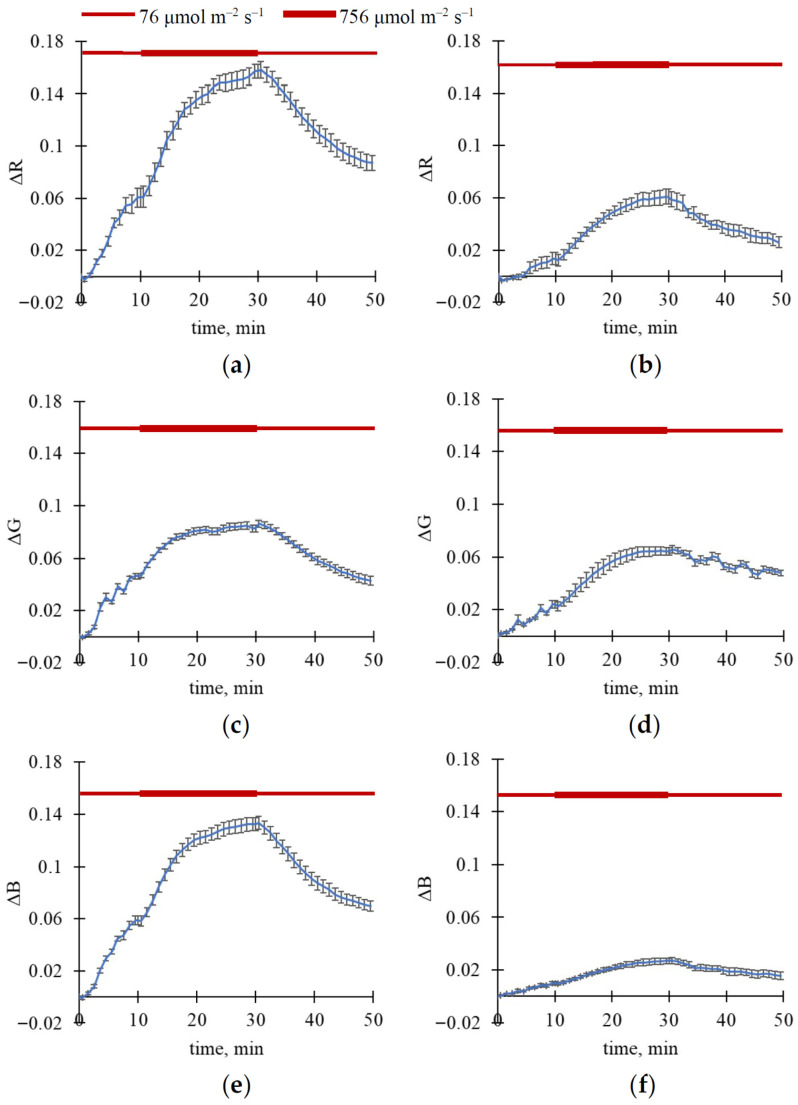
Dynamics of average ΔR, ΔG, and ΔB under three steps of light intensity (10 min for 76 μmol m^−2^ s^−1^, 20 min for 756 μmol m^−2^ s^−1^, and 20 min for 76 μmol m^−2^ s^−1^). ΔR in wheat (**a**) and pea (**b**), ΔG in wheat (**c**) and pea (**d**), and ΔB in wheat (**e**) and pea (**f**) are shown. A total of 12 wheat and 4 pea plants were analyzed (*n* = 12 and *n* = 4). A total of 24 ROIs were analyzed for wheat plants (*n* = 24), and 8 ROIs were analyzed for pea plants (*n* = 8). ΔR, ΔG, and ΔB were calculated for each light step as differences between values measured in the current time and those measured immediately after step initiation. Average values and standard errors are shown in the figure.

To exclude these ultra-fast changes, we only analyzed slow changes in RGB reflectance (Figure 3 and Figure 4); these changes were calculated for each light step as differences between values measured in the current time and those measured immediately after step initiation. Importantly, this procedure could not fully exclude possible subtle effects potentially resulting from changes in the exposure time; however, the described procedure minimized their influence.

It was shown that increasing light intensity from 76 to 756 μmol m^−2^ s^−1^ induced an additional increase in reflectance in R, G, and B spectral bands in both wheat and pea leaves; the effect was larger in wheat plants (Figure 3). In contrast, decreasing light intensity from 756 to 76 μmol m^−2^ s^−1^ induced relatively slow relaxation (>20 min) of ΔR, ΔG, and ΔB in the investigated plants (Figure 4).

Our results thus showed that illumination increased reflectance in all R, G, and B spectral bands for a period of minutes to tens of minutes; this response depended on light intensity and was reversible.

### 2.2. Light-Induced Changes in Parameters of Photosynthetic Light Reactions

Parameters of photosynthetic light reactions, including the effective quantum yield of photosystem II (YII) and non-photochemical quenching of chlorophyll fluorescence (NPQ), were measured simultaneously with RGB reflectance.

**Figure 5:**
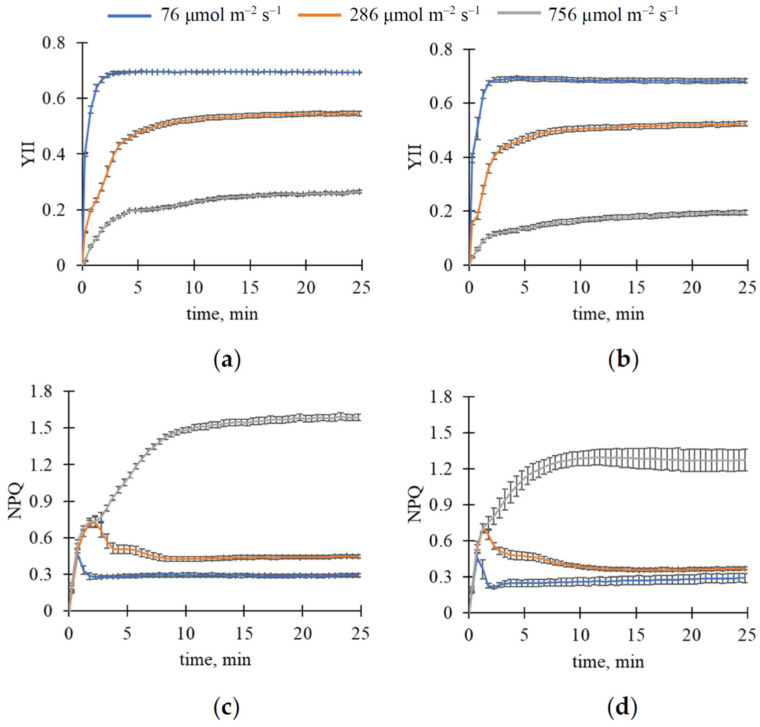
The influence of the 25 min illumination with constant light intensity (76, 286, and 756 μmol m^−2^ s^−1^) on the effective quantum yield of photosystem II (YII) and non-photochemical quenching of photosystem II (NPQ) in leaves. Dynamics of YII in wheat (**a**) and pea (**b**), and dynamics of NPQ in wheat (**c**) and pea (**d**) are shown. A total of 12 wheat and 6 pea plants were analyzed (*n* = 12 and *n* = 6). Average values and standard errors are shown in the figure.

Analysis of the maximal quantum yield of photosystem II, which was measured after 15 min of dark adaptation, showed that this parameter was 0.8063 ± 0.0008 and 0.8097 ± 0.0015 for wheat and pea leaves, respectively. The dynamics of YII and NPQ under light with constant intensity are shown in Figure 5. In all investigated cases, YII was strongly decreased immediately after initiation of illumination; after that, this parameter gradually increased to a stationary level (Figure 5a,b). Notably, YII rapidly increased to a high stationary level under low light intensity (76 μmol m^−2^ s^−1^); in contrast, this parameter slowly increased to a low stationary level under moderate (286 μmol m^−2^ s^−1^) and, in particular, high (756 μmol m^−2^ s^−1^) light intensity. Slow-increasing YII under high light intensity seemed to be an additional component of the response and was absent under low light intensity.

NPQ analysis showed that this parameter increased after initiation of illumination in both wheat and pea leaves (Figure 5c,d). However, there were two types of dynamics: NPQ remained at its maximum for several minutes after the illumination initiation under low (76 μmol m^−2^ s^−1^) or moderate (286 μmol m^−2^ s^−1^) light intensity and then decreased; NPQ increased over a longer period, with changes including fast and slow components, under high light intensity (756 μmol m^−2^ s^−1^).

**Figure 6:**
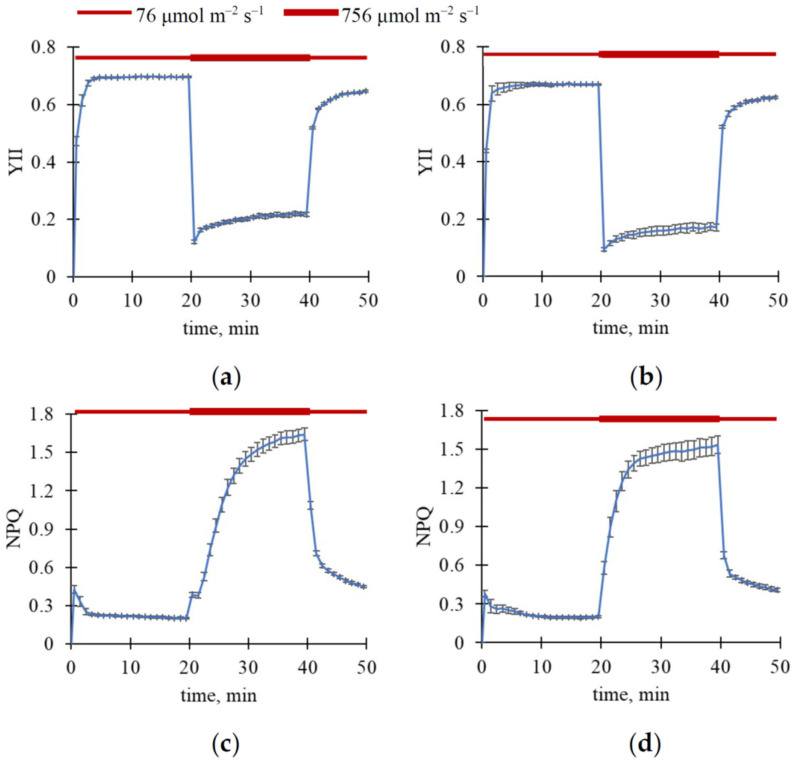
Dynamics of average YII and NPQ in leaves under three steps of light intensity (20 min for 76 μmol m^−2^ s^−1^, 20 min for 756 μmol m^−2^ s^−1^, and 10 min for 76 μmol m^−2^ s^−1^). Dynamics of YII in wheat (**a**) and pea (**b**), and dynamics of NPQ in wheat (**c**) and pea (**d**) are shown. A total of 11 wheat and 4 pea plants were analyzed (*n* = 11 and *n* = 4). Average values and standard errors are shown in the figure.

**Figure 7:**
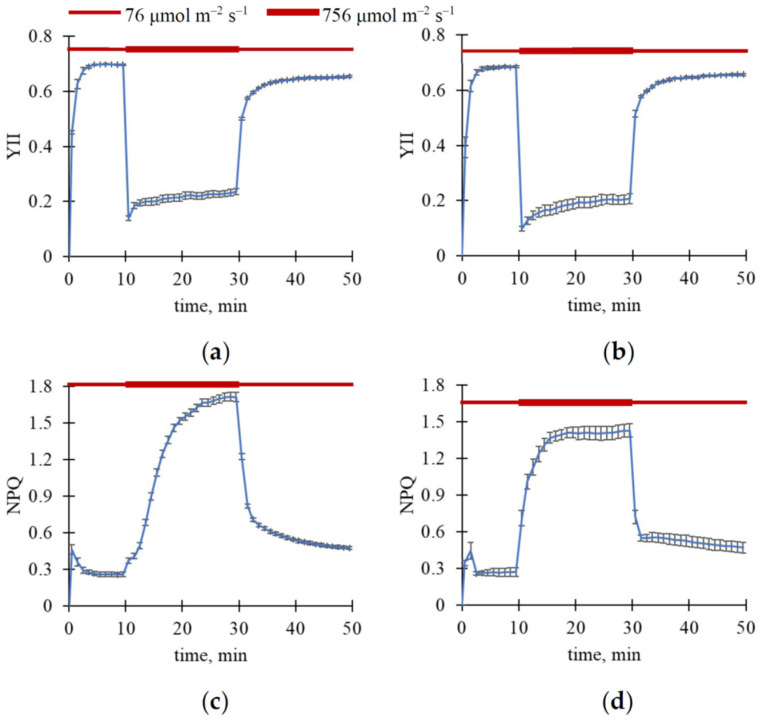
Dynamics of average YII and NPQ in leaves under three steps of light intensity (10 min for 76 μmol m^−2^ s^−1^, 20 min for 756 μmol m^−2^ s^−1^, and 20 min for 76 μmol m^−2^ s^−1^). Dynamics of YII in wheat (**a**) and pea (**b**), and dynamics of NPQ in wheat (**c**) and pea (**d**) are shown. A total of 12 wheat and 4 pea plants were analyzed (*n* = 12 and *n* = 4). Average values and standard errors are shown in the figure.

The increase in light intensity caused a decrease in YII and an increase in NPQ (Figure 6 and Figure 7). The following decrease in light intensity induced relaxation of these photosynthetic parameters. It should be noted that the slow component of changes in YII and, in particular, NPQ was observed after the increase in light intensity as well as after the decrease in this intensity.

Illumination thus induced changes in YII and NPQ with fast and slow components. Dynamics of the slow component of changes, which were maximal under high light intensity, seemed to be similar to those of ΔR, ΔG, and ΔB.

### 2.3. Analysis of Linear Correlations Between Parameters of RGB Reflectance and Photosynthetic Light Reactions

To determine how changes in reflectance in R, G, and B spectral bands relate to those in YII and NPQ, we performed correlation analysis between the investigated parameters. It was shown that Pearson correlation coefficients between parameters of RGB reflectance (ΔR, ΔG, and ΔB) and photosynthetic light reactions (YII and NPQ) were high under high light intensity and low under low light intensity (Table 3).

**Figure 8:**
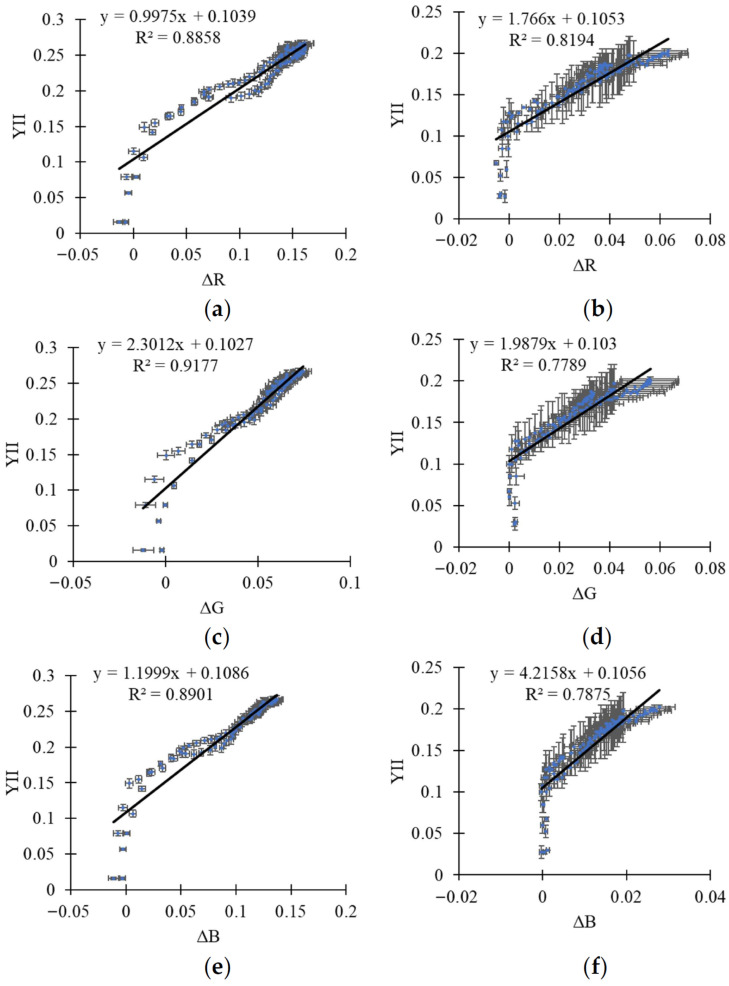
Scatter plots between parameters of RGB reflectance and parameters of photosynthetic light reactions under 756 μmol m^−2^ s^−1^ light intensity. The relations between ΔR and YII in wheat (**a**) and pea (**b**), between ΔG and YII in wheat (**c**) and pea (**d**), and between ΔB and YII in wheat (**e**) and pea (**f**) are shown. Average values and standard errors are shown in the figure.

The scattering plots between parameters of RGB reflectance (ΔR, ΔG, and ΔB) and YII under 756 μmol m^−2^ s^−1^ light intensity are shown in Figure 8. It was shown that linear regressions well described the relations between the investigated parameters. However, the figure also supports that photosynthetic changes comprised two components; only one of them (slow) was likely to be strongly and linearly related to ΔR, ΔG, and ΔB.

### 2.4. Model-Based Analysis of Influence of Chloroplast Localization on Reflectance and Effective Quantum Yield of Photosystem II in Leaf

A simple mathematical model of optical properties (based on the Kubelka–Munk theory) and photosynthetic light reactions (based on the Farquhar–von Caemmerer–Berry model) in leaves was developed to qualitatively analyze the potential influence of changes in chloroplast localization on RGB reflectance and YII (see Section 4.4 for details). It should be noted that using the qualitative model, which could not describe investigated processes in the quantitative manner, avoided excessive detail regarding the optical and photosynthetic description and minimized parameterization of the model; e.g., description of differences between mesophyll layers in wheat (the uniform mesophyll layer with moderate light scattering) and pea (the palisade mesophyll layer with high light absorption and low light scattering and spongy mesophyll layer with moderate light absorption and high light scattering) [19,42] was excluded.

**Figure 9:**
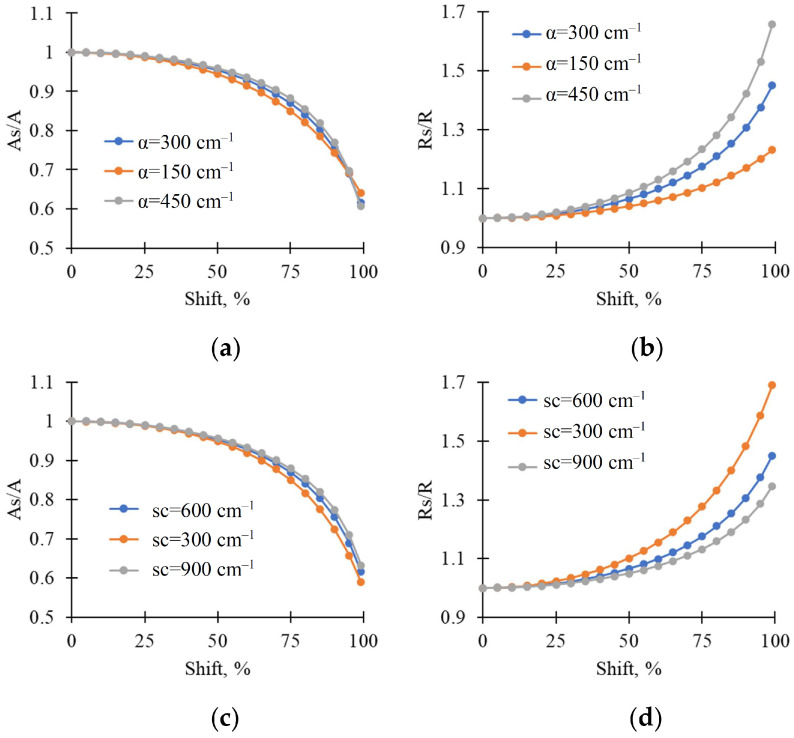
Model-based dependences of the normalized averaged light absorption (As/A) and reflectance (Rs/R) on heterogeneity in the linear light absorption coefficient (Shift). The heterogeneity, which was assumed as the minimal description of the light-induced heterogeneity in chloroplast localization, was described through using two regions of leaf with equal areas: the region with the decreased linear light absorption coefficient $\alpha_{1}=\alpha -\frac{shift}{100}\alpha$ (corresponded to the decreased chloroplast content in the model) and with the increased linear light absorption coefficient $\alpha_{2}=\alpha +\frac{shift}{100}\alpha$ (corresponded to the increased chloroplast content in the model); α is the average linear light absorption coefficient in leaf. Values of leaf absorption and reflectance were averaged over two regions. (**a**) Dependences of As/A on Shift at different α; (**b**) Dependences of Rs/R on Shift at different α; (**c**) Dependences of As/A on Shift at different sc; (**d**) Dependences of Rs/R on Shift at different sc. The averaged light absorption and reflectance were normalized using their values at Shift = 0% (absence of heterogeneity). Parameters of the model are shown in Section 4.4.

Model-based analysis showed (Figure 9) that increasing heterogeneity (Shift) in the linear light absorption coefficient (α), which was taken as the simplest description of the light-induced increase in heterogeneity in chloroplast localization, decreased the normalized averaged light absorption (As/A) and increased the normalized averaged light reflectance (Rs/R) in the leaf. In addition, modifications in the average value of the linear light absorption coefficient (Figure 9a) and value of the coefficient of light scattering (sc) (Figure 9c) weakly influenced the dependence of the averaged light absorption by leaf on heterogeneity in chloroplast localization. In contrast, increasing the average α (Figure 9b) and decreasing sc (Figure 9d) increased the dependence of the averaged light reflectance on heterogeneity in chloroplast localization.

**Figure 10:**
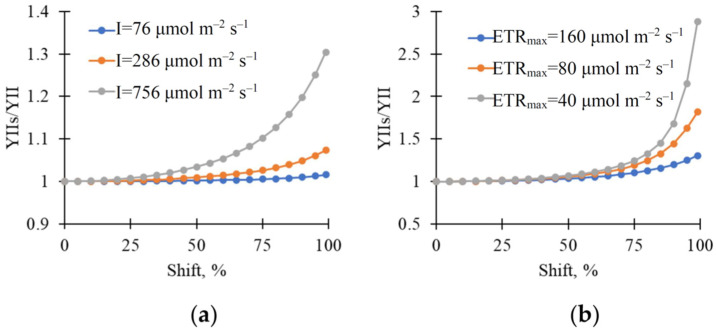
Model-based dependences of normalized averaged YII (YIIs/YII) on heterogeneity in the linear light absorption coefficient (Shift). (**a**) Dependence of YII on Shift under different light intensity; (**b**) dependence of YII on Shift at different ETR_max_ under 756 μmol m^−2^ s^−1^ light intensity. The averaged YII was normalized using its value in the leaf with a homogeneous distribution of chloroplasts (Shift = 0%). Parameters of the model are shown in Section 4.4.

Further, we analyzed the influence of the heterogeneity in chloroplast localization on the averaged YII. It was shown that normalized YII (YIIs/YII) was weakly dependent on Shift under low light intensity (Figure 10a); in contrast, this dependence was strongly increased under high light intensity. This dependence was further increased with decreased ETR_max_, which is the maximal electron transport rate in the photosystem II (Figure 10b).

Model-based analysis results were thus in good accordance with the hypothesis regarding the role of light-induced heterogeneity of chloroplast localization in simultaneously increasing RGB reflectance and effective quantum yield of photosystem II.

### 2.5. Analysis of Light-Induced Changes in Transmittance and Reflectance of Narrow-Band Blue Light in Leaves of Pea

The results of the model-based analysis showed that forming areas with increased and decreased linear light absorption coefficients, which were taken as the minimal description of chloroplast content heterogeneity, increased reflectance and YII. The responses were qualitatively similar to light-induced increases in these parameters in our experiments. As a result, it was hypothesized that the chloroplasts’ movement and changes in their localization were probably mechanisms forming light-induced changes in reflectance in R, G, and B spectral bands.

**Figure 11:**
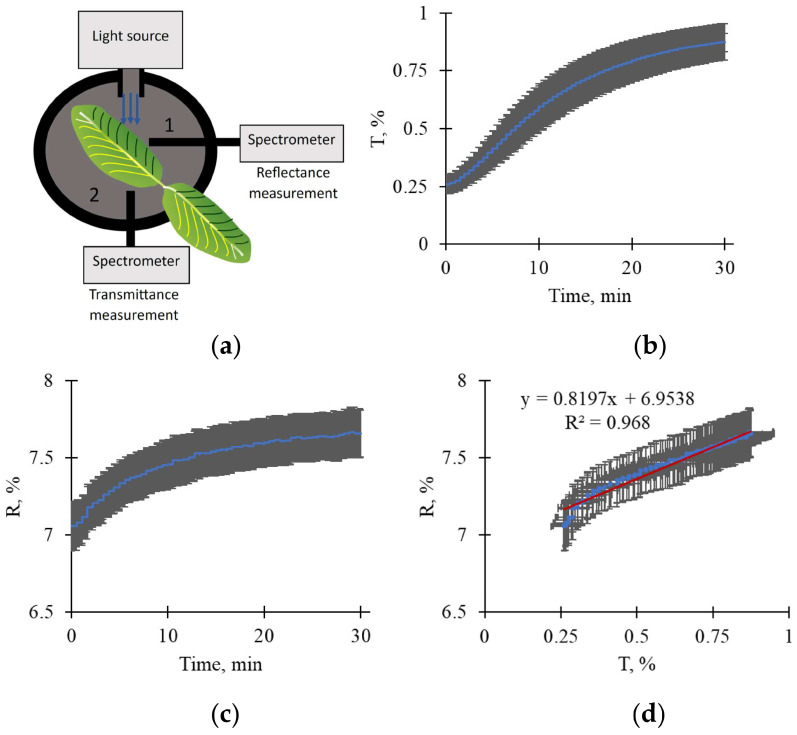
(**a**) The scheme for measuring transmittance and reflectance for narrow-band blue light in pea leaves. The light source with 460 nm maximum and 20 nm half-width (Measuring head DUAL-DB in DUAL-PAM-100 (Heinz Walz GmbH, Effeltrich, Germany)) and the spectrometer (FLAME-S-VIS-NIR (Ocean Optics, Orlando, FL, USA)) were used; they were positioned at an angle of 45° relative to the plant leaf. The reflectance and transmittance were measured in positions 1 and 2, respectively; different series of plants were used. The leaf reflectance and transmittance were averaged in the spectral range of 457–462 nm (mean was 460 nm). (**b**) The dynamics of average leaf transmittance at 758 μmol m^−2^ s^−1^ light intensity in pea leaves (*n* = 6). (**c**) The dynamics of average leaf reflectance at 758 μmol m^−2^ s^−1^ light intensity in pea leaves (*n* = 5). (**d**) The scatter plots between these transmittance and reflectance. Changes in transmittance and reflectance were initiated by the action of light with 758 μmol m^−2^ s^−1^ intensity (30 min) after adaptation under dark conditions for 15 min. Average values and standard errors are shown in the figure.

It is known that changes in light transmittance could be related to the light-induced movement of chloroplasts [11]. To additionally test the hypothesis regarding changes in chloroplast localization, we thus investigated the influence of light on transmittance and reflectance in pea leaves at 460 nm (Figure 11). The results showed that the action of light at 758 μmol m^−2^ s^−1^ light intensity increased both transmittance and reflectance of the leaf; both changes were strongly linearly related. The results were consistent with the hypothesis regarding the role of chloroplast movement in forming light-induced ΔR, ΔG, and ΔB in leaves of the investigated plants.

## 3. Discussion

In plants, light performs important functions, such as supplying energy, regulating differentiation and development, and inducing phototropism and adaptive responses [2,3,4]. The influence of light on plants often relates to pigments that participate in different metabolic and regulatory processes, including stress changes [10,17]. It is known that pigments mostly absorb light in the visible spectral region [8,9]; as a result, the parameters of visible light, which are reflected or transmitted by leaves, can be used to analyze characteristics of plants under different environmental conditions [9,21].

RGB imaging is an available method for measuring parameters of light reflectance in the visible spectral region [34]. It is often applied to estimate plant parameters such as biomass, chlorophyll content, and nitrogen content [29,30,31,32]. Changes in these parameters are slow (from days to months or more) [28,34]. However, parameters of RGB reflectance can potentially change over short time intervals (from minutes to hours) through fast changes in light reflectance and absorption in leaves; specifically, we showed relatively fast changes in broadband reflectance (tens of minutes) induced by propagation of electrical signals [36].

It is known that fluctuations in light intensity (particularly in the minute range) are widely observed in natural conditions and strongly influence plant photosynthetic processes [43,44]. As a result, this study investigates fast changes in RGB reflectance caused by illumination with different light intensities. Our results show that illumination increases reflectance in R, G, and B spectral bands (Figure 1, Figure 2, Figure 3 and Figure 4); the main changes are formed for about 10 min in wheat leaves and about 15–20 min in pea leaves. Analysis of magnitudes of ΔR, ΔG, and ΔB (Table 1, Figure 2) shows that light intensity influences changes in RGB reflectance in wheat plants; in contrast, this influence is not shown in pea plants in the investigated range of light intensities. On the other hand, increasing the light intensity after low-intensity illumination additionally increases RGB reflectance in both wheat and pea leaves (Figure 3 and Figure 4); decreasing the light intensity decreases this reflectance. Our results also show that the light-induced increase in RGB reflectance is higher in wheat leaves than in pea leaves (Figure 2, Table 2).

The current analysis thus shows that reflectance in R, G, and B spectral bands can be changed, at a minimum, in the minute range; i.e., RGB reflectance is sensitive to relatively fast plant responses to changes in illumination under controlled conditions. Potential mechanisms for driving these changes in RGB reflectance require additional consideration. It is unlikely these mechanisms are related to fast light-induced changes in leaf optical properties caused by transition in the xanthophyll cycle [45,46,47], chloroplast shrinkage [14,15,48], or electrochromic shift [49] because these processes modify plant transmittance and reflectance in green spectral regions. In contrast, changes in leaf water content, which can influence reflectance of visible light in accordance with our earlier investigations [35,36], are gradual and relatively slow (>25 min); their involvement in increasing RGB reflectance, which reaches 50% for about 5 min in wheat leaves (see, e.g., Figure 1), does not seem to be probable.

Simultaneous decreases in concentrations of basic photosynthetic pigments (chlorophylls and carotenoids) can potentially increase RGB reflectance in plant leaves because they absorb visible light in all spectral bands (with different efficiency) [50,51]. On the other hand, changes in concentrations of chlorophylls and carotenoids are slow and can be accompanied by changes in the ratio between concentrations of these pigments [10]. It means that direct changes in the pigment concentrations are unlikely to influence the demonstrated light-induced changes in RGB reflectance.

However, it is well known that light action induces changes in chloroplast localization in the plant mesophyll cell to form regions in this cell with increased and decreased content of chloroplasts (and, therefore, photosynthetic pigments) [38]. The changes in chloroplast localization decrease light absorption via chlorophylls and prevent damage to the photosynthetic apparatus [13]; i.e., they decrease average light absorption and increase average reflectance and transmittance in leaves [11]. Light-induced changes in chloroplast localization can be formed in the minute range [11,37,52,53,54]; specifically, the duration required for 50% changes in chloroplast localization can vary from about 5–6 min (e.g., in Arabidopsis [52,53] or barley [54]) to about 20 min or more (e.g., in *Alocasia macrorrhiza* [11]).

As a result, we hypothesized that the light-induced chloroplast movement can be a mechanism of light-induced increases in RGB reflectance, as shown in this study.

There are several indirect arguments that support the hypothesis:

- It is known that light-induced chloroplast movement can decrease light absorption and increase reflectance in all spectral regions of visible light [11]. A light-induced increase in RGB reflectance, as shown in this study (Figure 1, Figure 2, Figure 3 and Figure 4), is in accordance with this point.
- It is known that changes in chloroplast localization under illumination are formed in the minute range; specifically, the duration necessary for 50% changes can be about 5–6 min for Arabidopsis [52,53] or barley [54], about 9 min for tobacco [54], or more for others. In this study, we show that durations required for a 50% increase in RGB reflectance under illumination are mainly about 4.5–6.5 min in wheat leaves and 7–9.5 min in pea leaves (see Figure 1); i.e., changes in RGB reflectance (our experiments) and changes in chloroplast localization (literature data) can exhibit similar durations.
- Analysis of the developed qualitative model, based on the Kubelka–Munk theory widely used to analyze leaf optical properties [55,56,57], shows that the model describes increasing reflectance and decreasing absorption as heterogeneity in the linear light absorption coefficient is increased (Figure 9). Considering that this increase in α heterogeneity is the simplest description for the increase in chloroplast localization heterogeneity, the model-based result is in accordance with the hypothesis regarding participation of the light-induced chloroplast movement in increasing RGB reflectance.
- Light-induced changes in leaf light transmittance are widely used as an indirect indicator of chloroplast movement under illumination [11,52,53,54]; specifically, transmittance in the blue spectral region can be increased by at least 250–300% [11,54]. Our results show that illumination increases this blue light transmittance in pea leaves by about 250% (Figure 11b); this increase in light transmittance is strongly linearly related to reflectance (Figure 11d). The result additionally supports the hypothesis regarding the participation of the light-induced chloroplast movement in increasing RGB reflectance (at least, for pea plants).

In addition, the proposed hypothesis is potentially in accordance with differences between magnitudes of responses in wheat and pea leaves (Figure 2). It is known that monocot plants (including wheat) mainly exhibit a uniform mesophyll with moderate light scattering; in contrast, dicot plants (including pea) mainly have a palisade mesophyll with high light absorption and low light scattering, and a spongy mesophyll with moderate light absorption and high light scattering [19,20,42]. Considering this point, it can be expected that adaxial leaf surface reflectance in wheat leaves (with moderate light scattering in mesophyll) should be higher than that in pea leaves (with low light scattering in the palisade mesophyll) due to the high scattering-related reflectance component; the statement also seems to be correct for changes in reflectance. Another component of the total reflectance is surface reflectance, which is caused by surface roughness and is weakly related to internal leaf structure [20,42]; changes to surface reflectance in the minute range are not probable. These points are in good agreement with the low initial RGB reflectance (see Section 2.1) and low magnitude of its light-induced changes (Figure 2) in pea leaves in comparison to those in wheat leaves, as shown in this study.

Our results also highlight that the slow components of increasing YII and NPQ under high light intensity (Figure 5, Figure 6 and Figure 7) seem to have a similar rate of change to the increasing RGB reflectance (Figure 1, Figure 3 and Figure 4); values of YII and NPQ are strongly correlated with ΔR, ΔG, and ΔB under these light conditions (Table 3). In contrast, these correlations are mainly weak under low and moderate light intensities (Table 3). Correlations cannot strictly show causal relationships; specifically, it cannot be ruled out that correlations can be observed even in the absence of a direct mechanistic linkage, because RGB reflectance and photosynthetic parameters are affected by the same light action. However, if the hypothesis regarding participation of the light-induced chloroplast movement in increasing RGB reflectance (see above) is correct, then changes in both RGB reflectance and photosynthetic parameters can be attributed to that movement.

It is known that light-induced changes in the localization of chloroplasts, which decrease the average light absorption [11], can induce an apparent increase in NPQ [39,40,41]. This effect is caused by decreasing absorption of the measuring light, which is used to measure NPQ and other photosynthetic parameters via pulse-amplitude-modulated (PAM) fluorometry [58]; i.e., it does not directly show a real increase in light energy dissipation or real damage of photosynthetic machinery, which are typically related to forming NPQ [59]. Importantly, the relative contribution of chloroplast movement to total NPQ dynamics can increase with increasing light intensity [40]; the fast energy-dependent changes in NPQ likely mask this component under lower light intensity. Our results are in good accordance with these points: (i) there is slow component of NPQ increasing under high light intensity (Figure 5c,d), and NPQ is strongly correlated with RGB reflectance under these light conditions (Table 3); (ii) changes in NPQ have a complex shape under low and moderate light intensity, and NPQ is weakly correlated with RGB reflectance.

The effective quantum yield of photosystem II is only calculated on the basis of the fluorescence parameters measured at the same time [58], which means that light-induced decreases in leaf absorption cannot influence YII through changes in the intensity of the measured light. Importantly, light-induced chloroplast movement should also decrease the absorption of actinic light [11]. If YII increases with decreasing actinic light intensity [60], it can be expected that decreasing this light absorption should also increase YII; moreover, the magnitude of the response should decrease with decreasing light intensity and increasing initial YII (the effective quantum yield cannot be higher than the maximal quantum yield of photosystem II). Our model-based analysis revealed similar effects: increasing the chloroplast localization heterogeneity has a strong effect on YII under high light intensity and a weak one under low light intensity (Figure 10a). The current experimental results, which show that increasing RGB reflectance and YII are strongly and positively correlated under high light intensity and weakly correlated under low light intensity (Table 3, Figure 8), are in good agreement with these points.

It is interesting that the model-based analysis also shows that decreasing the maximal electron flow through the photosynthetic electron transport chain (ETR_max_) stimulates the effect of heterogeneity in chloroplast distribution on YII simulated by the model (Figure 10b). It is known that stressors often cause limitations in ETR_max_ [61,62]; i.e., it cannot be ruled out that relations between light-induced changes in RGB reflectance and photosynthetic parameters can be stimulated under stressor action.

As a result, the hypothesis regarding the participation of light-induced chloroplast movement in increasing RGB reflectance is consistent with the high correlation coefficients between RGB reflectance and photosynthetic parameters under high light intensity and low coefficients under low light intensity, as well as with the model-based results.

**Figure 12:**
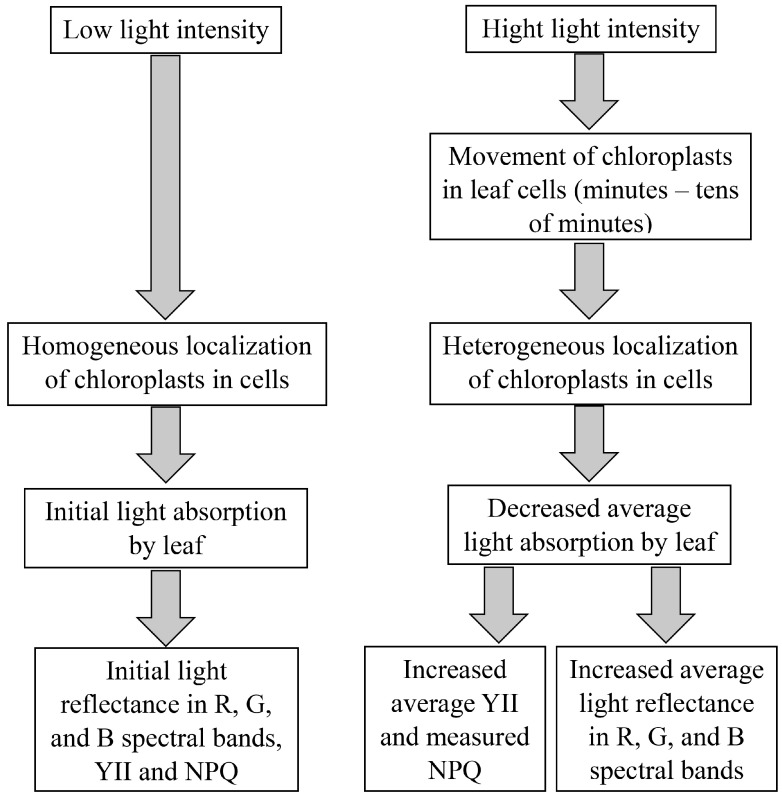
The hypothetical scheme of potential mechanisms of light influence on RGB reflectance and parameters of photosynthetic light reactions in the minute range. In accordance with the proposed hypothesis, the low light intensity weakly influences the localization of chloroplasts, resulting in their homogeneous distribution in leaf cells. Increasing light intensity induces movement of chloroplasts in leaf cells, resulting in a heterogeneous distribution. This heterogeneous distribution of chloroplasts decreases the average light absorption in leaves to minimize photodamage to photosynthetic machinery. Decreasing the average light absorption can increase the RGB reflectance and quantum yield of photosystem II (YII). Additionally, this decrease can increase the measured non-photochemical quenching of chlorophyll fluorescence (NPQ) by decreasing the absorption of light measured at NPQ using pulse-amplitude-modulated (PAM) fluorometry.

Thus, our results show that RGB reflectance is sensitive to changes in light intensity because increasing the intensity increases reflectance in the R, G, and B spectral bands in the minute range; in contrast, decreasing the light intensity causes relaxation of reflectance in these bands. Slow changes in RGB reflectance are strongly correlated with NPQ and YII under high light intensity; these correlations are low under low light intensity. Using qualitative model-based analysis, experimental investigation of the dynamics of blue light transmittance through pea leaves, and data from the literature, we hypothesized that changes in RGB reflectance and the parameters of photosynthetic light reactions are related to light-induced changes in chloroplast localization, resulting in heterogeneity in their distribution in leaf cells. The hypothetical scheme of mechanisms of these light-induced changes is illustrated in Figure 12. The proposed hypothesis seems probable because it is a simple explanation of all changes in RGB reflectance and photosynthetic parameters and the correlations between these changes, which are shown in the current work. However, other factors that may contribute to these effects cannot be fully excluded.

Finally, it should be noted that only wheat (a monocot plant) and pea (a dicot plant) were investigated in the current work. Responses in these plants were qualitatively similar; however, their quantitative characteristics (e.g., magnitude or duration of light-induced changes in RGB reflectance) differed. This means that analyzing the quantitative characteristics of light-induced changes in RGB reflectance and their relations to photosynthetic parameters in different plant species is an important task for future investigations. Additionally, it cannot be ignored that specific growth conditions also quantitatively influence light-induced changes in RGB reflectance.

## 4. Materials and Methods

### 4.1. Plant Materials and Cultivation

Pea (*Pisum sativum* L., cultivar “Falyonsky Yubileyniy”) and wheat *(Triticum aestivum* L., cultivar “Daria”) plants were cultivated in a vegetation room under controlled light and temperature conditions (16/8 day/night photoperiod; 23 °C). Daylight was provided by luminescent lamps FSL YZ18RR (Foshan Electrical And Lighting Co., Ltd., Foshan, China).

Plants were cultivated in pots with the peat soil “Morris Green” (Pelgorskoe M, Ryabovo, Russia); each pot contained 9 plants. The plants were irrigated three times per week with 50 mL of water. 2–3-week-old pea and wheat plants (early vegetative stages) were used in experiments.

### 4.2. Measuring RGB Reflectance and Photosynthetic Parameters and Data Processing

Open FluorCam FC 800-O/1010-S (Photon system instruments, Drásov, Czech Republic) was used to measure the effective quantum yield of photosystem II (YII) and non-photochemical quenching of chlorophyll fluorescence (NPQ).

(1) $$YII=\frac{F_{m}^{\prime }-F}{F_{m}^{\prime }},$$

(2) $$NPQ=\frac{{F_{m}-F}_{m}^{\prime }}{F_{m}^{\prime }},$$

where $F_{m}$ is the maximal quantum fluorescence yield of photosystem II in dark-adapted plants; $F_{m}^{\prime }$ is the maximal quantum fluorescence yield under light conditions; F is the fluorescence yield under illumination by the actinic light. Photosynthetic parameters were calculated for different ROIs (Figure S3b) using Open FluorCam FC 800-O/1010-S software; two ROIs per plant were analyzed.

The plants were allowed 15 min to adapt to dark conditions. White FluorCam LED panels provided actinic light during the measurements. We used the following plant illumination regimes:

(1) Intensity of 76 μmol m^−2^ s^−1^ (25 min); saturation pulses were turned on every 30 s.
(2) Intensity of 286 μmol m^−2^ s^−1^ (25 min); saturation pulses were turned on every 30 s.
(3) Intensity of 756 μmol m^−2^ s^−1^ (25 min); saturation pulses were turned on every 30 s.
(4) The first illumination regime, with three steps (more suitable for achieving initial stationary levels of RGB reflectance under the 76 μmol m^−2^ s^−1^ light intensity) as follows: 76 μmol m^−2^ s^−1^ (20 min) => 756 μmol m^−2^ s^−1^ (20 min) => 76 μmol m^−2^ s^−1^ (10 min). The saturation pulses were turned on every 60 s.
(5) The second illumination regime, with three steps (more suitable for achieving final relaxation of RGB reflectance under the 76 μmol m^−2^ s^−1^ light intensity), was as follows: 76 μmol m^−2^ s^−1^ (10 min) => 756 μmol m^−2^ s^−1^ (20 min) => 76 μmol m^−2^ s^−1^ (20 min). The saturation pulses were turned on every 60 s.

Photosynthetic parameters and RGB reflectance were simultaneously measured. The locations of the devices and plants are shown in Figure S3a, and the plants and devices were darkened with a black cloth.

A Canon EOS 4000D (Canon Inc., Tokyo, Japan) camera was used as the RGB camera. The distance between the camera lens and the plants was 45 cm, and the focus length was 35 mm. ISO was 100. The “tungsten” white balance was used (automatic white balance was turned off), in accordance with our previous work [24].

To optimize the parameters of the RGB measurements, we measured the values of color parameters according to the white reflectance standard (Qp Card 101.3, Argraph Corp., Carlstadt, NJ, USA), using a series of exposures with RGB camera (1/10, 1/20, 1/30, 1/40, 1/50, 1/60, 1/80, 1/100, 1/125, 1/160, 1/200, 1/250, 1/320, 1/400, 1/500, 1/640, 1/800, 1/1000, 1/1250, 1/1600, 1/2000, 1/2500, 1/3200, and 1/4000 s) under each of the five light levels (76, 286, 412, 581, and 756 μmol m^−2^ s^−1^). These values are shown in Figure S4 as measured values in the R, G, and B spectral channels; only values ≤ 250 arbitrary units were analyzed to minimize errors.

The RGB camera can capture intensity in a range from 0 to 255 arbitrary units. In contrast, the real intensities of objects can be higher than 255; these intensities were calculated based on exposure times. To calculate these real intensities, the exposure time, which provided a measured intensity of approximately 100 arbitrary units, was selected for each light intensity; this time was assumed to be 100%. Other real intensities (for this light intensity and for different exposures) were calculated proportionally. The relationships between the measured and calculated values (Figure S4) were fitted well by linear (<140–180 arbitrary units) and exponential (>140–180 arbitrary units) regressions. It should be noted that the linear regressions had coefficients equaling approximately 1. The results showed that the measured and calculated (real) values of the light intensity were approximately the same at values < 140 arbitrary units for all spectral channels.

Considering this result, we selected exposure times for the RGB camera of 1/40 s for 76 μmol m^−2^ s^−1^, 1/160 s for 286 μmol m^−2^ s^−1^, and 1/400 s for 756 μmol m^−2^ s^−1^. These exposures provided values of all measured light intensities lower than 140 arbitrary units (approximately 20–120 for leaves and 40–100 for the gray reflectance standard Qp Card 101.3 in different spectral channels); i.e., all measured values of the reflected light intensity were linearly related to the real ones. It should be noted that we used the 18% gray reflectance standard to calculate the normalized reflectance because its total light reflectance value is similar to that of plant leaves (Figure S5). Using the measured intensities of the reflected light on the white and gray standards and scatter plots between the measured and real intensities (Figure S4), we calculated the ratio of the reflectance of the gray and white standards, which was about 0.23.

The following experimental procedure was used to measure RGB reflectance. Measurements of a series of 5 photos were initiated in 1–2 s after each saturation pulse by the RGB camera. Measurements of an additional series of 5 photos were taken 1–2 s after the initiation of illumination and, if applicable, after each change in intensity of the actinic light (before saturation pulses). The total duration of the series was about 4 s. The parameters of plant RGB reflectance in 5 photos of one series did not differ; i.e., there were no changes in the optical properties of the leaves in this time interval (from 1 to 2 s after saturation pulse or changing light intensity to 5–6 s after that).

The photos were processed using ImageJ 1.53e. Each photo was split into images according to the R, G, and B spectral channels. The values of reflected light in the R, G, and B spectral channels, which were measured in plant leaves, were averaged for each ROI (Figure S3b); two ROIs per plant were analyzed. The gray reflectance standard (see Figure S3a) was used for signal normalization in the R, G, and B channels. The normalized R, G, and B signal values were averaged for each series of photos (5 photos) to decrease signal noise.

Average values of RGB reflectance were calculated using an additional series of 5 photos, which were measured in dark-adapted plants immediately after turning on the light, and were used as initial zero levels in all experimental cases. In cases with changes in light intensity from 76 to 756 μmol m^−2^ s^−1^ and vice versa, the average values of RGB reflectance, which were measured immediately after changes in the light intensity (additional series of 5 photos), were used as additional zero levels for new light steps to exclude ultra-fast changes in measured reflectance (<several seconds).

Finally, normalized reflectance in the R, G, and B spectral bands, YII, and NPQ in pairs of ROIs, which were placed on leaves in the same plant, were also averaged; i.e., reflectance and photosynthetic parameters in separate plants were used as independent repetitions.

### 4.3. The Leaf Reflectance and Transmittance Measurement

Light-induced changes in the transmittance and reflectance of narrow-band blue light (with 460 nm maximum and 20 nm half-width) in pea leaves were measured to further support the hypothesis about the participation of light-induced chloroplast movement in increasing RGB reflectance. It is known that light-induced changes in leaf light are widely used indirect indicators of chloroplast movement under illumination [11,52,53,54,63,64]; these changes are maximal for blue light [11].

An optical unit for suspensions, ED-101US/MD in DUAL-PAM-100 (Heinz Walz GmbH, Effeltrich, Germany), was used as the basis of the setup for fixed localization of pea leaves and for measurements of transmittance and reflectance (Figure 11a). The measuring head DUAL-DB in DUAL-PAM-100 (Heinz Walz GmbH) was used as the source of narrow-band blue light with regulated intensity and duration. A spectrometer FLAME-S-VIS-NIR (Ocean Optics, Orlando, FL, USA) with a fiber optic cable and collimator (light input) was used to measure light intensity, which was averaged in the spectral range 457–462 nm (mean: 460 nm). The spectrometer had a 350–1000 nm spectral range and 0.374 nm spectral resolution; a 50 ms integration time was used.

The intensities of reflected and transmitted light were measured at positions 1 and 2, respectively (Figure 11a); different series of plants were used. Both the light source and light input of the spectrometer were positioned at an angle of 45° relative to the surface of the pea leaf. The intensity of light transmitted without leaves was assumed to be 100% when calculating transmittance. The intensity of light reflected from the white standard was assumed to be 100% when calculating reflectance.

Measurements of the reflectance and transmittance of pea leaves were initiated after 15 min of dark adaptation. A 758 μmol m^−2^ s^−1^ blue light intensity was used to induce changes in chloroplast localization; the total duration of illumination was 30 min.

### 4.4. Qualitative Mathematical Model of Optical Properties and Photosynthetic Activity in Leaves

We developed a qualitative mathematical model that included minimal descriptions of the optical properties of the plant leaf and photosynthetic activity. The leaf was described as a thin plate that scattered and absorbed light. The distribution of light in the leaf was described using the Kubelka–Munk theory for two light flows (Figure S6a):

(3) $$\begin{array}{c} \frac{dI}{dx}=-\left(\alpha +sc\right)\cdot I+sc\cdot J\\ \frac{dJ}{dx}=-sc\cdot I+\left(\alpha +sc\right)\cdot J \end{array}$$

where I is the forward light flow in the lamina; J is the backward light flow in the lamina; α is the linear light absorption coefficient (300 cm^−1^ in the basic variant [65]); sc is the coefficient of light scattering (600 cm^−1^ in the base variant [65]); x is the distance from the adaxial leaf surface.

Assuming zero illumination of the abaxial leaf surface, the equations in (3) were solved via Euler’s method of undetermined coefficients:

(4) $$\begin{array}{c} I\left(x\right)=A_{1}e^{\lambda x}+A_{2}e^{-\lambda x}\\ I\left(x\right)=B_{1}e^{\lambda x}+B_{2}e^{-\lambda x}\\ \lambda =\sqrt{\alpha^{2}+2\cdot \alpha \cdot sc}\\ A_{1}={-I}_{0}\cdot \frac{\alpha +sc-\lambda }{\left(\alpha +sc+\lambda \right)\cdot e^{2\cdot h\cdot \lambda }-(\alpha +sc-\lambda )}\\ A_{2}=I_{0}\cdot \frac{(\alpha +sc+\lambda )\cdot e^{2\cdot h\cdot \lambda }}{\left(\alpha +sc+\lambda \right)\cdot e^{2\cdot h\cdot \lambda }-(\alpha +sc-\lambda )}\\ B_{1}={-I}_{0}\cdot \frac{sc}{\left(\alpha +sc+\lambda \right)\cdot e^{2\cdot h\cdot \lambda }-(\alpha +sc-\lambda )}\\ B_{2}=I_{0}\cdot \frac{sc\cdot e^{2\cdot h\cdot \lambda }}{\left(\alpha +sc+\lambda \right)\cdot e^{2\cdot h\cdot \lambda }-(\alpha +sc-\lambda )} \end{array}$$

where I_0_ is the light intensity in the leaf near the adaxial surface. We did not consider reflectance, which relates to “leaf–air” or “air–leaf” boundaries on the leaf surface. As a result, I_0_ was assumed to be the incident light (the light from the light source).

The reflectance (R), transmittance (T), and absorption (A) of the leaf were described as follows:

(5) $$\begin{array}{c} R=\frac{sc\cdot (e^{2\cdot h\cdot \lambda }-1)}{\left(\alpha +sc+\lambda \right)\cdot e^{2\cdot h\cdot \lambda }-(\alpha +sc-\lambda )}\\ T=\frac{2\cdot \lambda \cdot e^{2\cdot h\cdot \lambda }}{\left(\alpha +sc+\lambda \right)\cdot e^{2\cdot h\cdot \lambda }-(\alpha +sc-\lambda )}\\ A=1-R-T \end{array}$$

where h (0.01 cm [65]) is the leaf thickness.

We assumed that light-induced chloroplast movement can be minimally described as increasing heterogeneity in leaf absorption properties because chloroplast movement should increase the linear light absorption coefficient in regions with increased chloroplast content and decrease this coefficient in regions with decreased chloroplast content (Figure S6b). Additionally, assuming that these regions had similar areas, we estimated the average light absorption under heterogeneous optical properties (A_s_) as follows:

(6) $$\begin{array}{c} A_{s}=\frac{A(\alpha_{1})+A(\alpha_{2})}{2}\\ \alpha_{1}=\alpha -\frac{Shift}{100}\alpha \;\mathrm{and}\;\alpha_{2}=\alpha +\frac{Shift}{100}\alpha \end{array}$$

where A(α_1_) is the light absorption in a region with decreased chloroplast content, A(α_2_) is the light absorption in a region with increased chloroplast content, α is the average linear light absorption coefficient, and α_1_ and α_2_ are linear light absorption coefficients in regions with decreased and increased chloroplast contents, respectively. Shift is the change in the linear light absorption coefficient in comparison to the initial value.

The averaged reflectance under heterogeneous optical properties (R_s_) was described as

(7) $$R_{s}=\frac{R(\alpha_{1})+R(\alpha_{2})}{2}$$

where R(α_1_) and R(α_2_) are leaf reflectances in regions with decreased and increased chloroplast contents, respectively.

The activity of photosynthetic light reactions was described using equations from the Farquhar–von Caemmerer–Berry model [5]:

(8) $$\begin{array}{c} ETR=\frac{I_{2}+{ETR}_{max}-\sqrt{{\left(I_{2}+{ETR}_{max}\right)}^{2}-4\cdot \theta \cdot I_{2}\cdot {ETR}_{max}}}{2\cdot \theta }\\ I_{2}=0.5\cdot I\cdot A\cdot (1-f) \end{array}$$

where ETR is the current flow through the photosynthetic electron-transported chain under the specific light intensity; ETR_max_ is the maximum flow the through the photosynthetic electron-transported chain (160 μmol m^−2^ s^−1^ in the basic variant [5]); I_2_ is the light intensity used in photosystem II; θ is the empirical curvature factor (0.7; in accordance with [5]); 0.5 is the portion of light absorbed by photosystem II; and f is the spectral quality of the light (0.15 [5]). The quantum yield of photosystem II (YII) was described as follows:

(9) $$YII=\frac{ETR}{I_{2}}$$

The averaged YII under heterogeneous optical properties (YII_s_) was calculated by averaging YII values in regions with decreased chloroplast content (YII(α_1_)) and in regions with increased chloroplast content (YII(α_2_)):

(10) $${YII}_{s}=\frac{YII\left(\alpha_{1}\right)+YII\left(\alpha_{2}\right)}{2}$$

### 4.5. Statistics

Quantities of ROIs are shown in figures; there were two ROIs per wheat or pea plant. The normal distribution of investigated values was confirmed using the Shapiro normality test. The homogeneity of variance was confirmed using Levene’s test; as a result, parametric statistics were used.

Average values, standard errors, and scatter plots are shown. Pearson correlation coefficients were used to estimate relations between investigated values. Determination coefficients were used to estimate the accuracy of linear regressions. The two-way ANOVA was used to analyze the influence of light intensity and plant species on ΔR, ΔG, and ΔB. Tukey’s HSD test was used to analyze the significance of differences between the groups investigated.

Analyses were performed using Microsoft Excel 2016 and Python 3.13.9 (scipy.stats, statsmodels, pandas).

## Figures and Tables

**Table 1:** Results (*p* values) of two-way ANOVA on magnitudes of ΔR, ΔG, and ΔB. Influences of light intensity, plant species, and the interaction between these factors were analyzed. Magnitudes were calculated as maximal ΔR, ΔG, and ΔB.

	Magnitude of ΔR	Magnitude of ΔG	Magnitude of ΔB
Light intensity	1.32 × 10^−8^	6.59 × 10^−10^	1.56 × 10^−12^
Plant species	7.06 × 10^−18^	3.17 × 10^−8^	1.37 × 10^−21^
Interaction of the factors	2.14 × 10^−4^	2.36 × 10^−4^	8.37 × 10^−6^

**Table 2:** Relative magnitudes of ΔR, ΔG, and ΔB under the action of light with different intensities in wheat and pea plants. Initial absolute values of reflectance in R, G, and B spectral bands were assumed as 100%.

		Wheat			Pea	
	ΔR	ΔG	ΔB	ΔR	ΔG	ΔB
76 μmol m^−2^ s^−1^	15.3 ± 0.7%	5.2 ± 0.2%	14.0 ± 0.7%	13.1 ± 1.9%	5.9 ± 1.1%	7.9 ± 1.9%
286 μmol m^−2^ s^−1^	20.0 ± 0.8%	8.2 ± 0.3%	22.3 ± 0.8%	8.6 ± 1.5%	4.6 ± 0.6%	8.5 ± 1.8%
756 μmol m^−2^ s^−1^	21.0 ± 0.6%	5.5 ± 0.2%	23.3 ± 0.6%	11.1 ± 1.6%	4.7 ± 0.8%	7.5 ± 1.0%

**Table 3:** Pearson correlation coefficients between average parameters of RGB reflectance (ΔR, ΔG, and ΔB) and photosynthetic light reactions (YII and NPQ). Values from Figure 1, Figure 3, Figure 4, Figure 5, Figure 6 and Figure 7 were used. Correlation coefficients with absolute values more than 0.7 are marked in bold. *, correlation coefficient is significant (*p* < 0.05).

	RGB Parameter	Wheat		Pea	
		YII	NPQ	YII	NPQ
76 μmol m^−2^ s^−1^	ΔR	0.518 *	−0.235 *	0.228 *	−0.188
	ΔG	0.538 *	−0.169	0.201 *	−0.441 *
	ΔB	0.506 *	−0.193	0.136	−0.470 *
286 μmol m^−2^ s^−1^	ΔR	0.843 *	−0.349 *	0.747 *	−0.616 *
	ΔG	0.866 *	−0.379 *	0.636 *	−0.560 *
	ΔB	0.846 *	−0.386 *	0.694 *	−0.567 *
756 μmol m^−2^ s^−1^	ΔR	0.941 *	0.979 *	0.905 *	0.726 *
	ΔG	0.958 *	0.970 *	0.883 *	0.647 *
	ΔB	0.943 *	0.977 *	0.887 *	0.636 *
76 (20 min), 756 (20 min), and 76 (10 min) μmol m^−2^ s^−1^	ΔR	−0.528 *	0.767 *	−0.523 *	0.678 *
	ΔG	−0.471 *	0.660 *	−0.417 *	0.571 *
	ΔB	−0.529 *	0.755 *	−0.479 *	0.631 *
76 (10 min), 756 (20 min), and 76 (20 min) μmol m^−2^ s^−1^	ΔR	−0.428 *	0.681 *	−0.384 *	0.590 *
	ΔG	−0.541 *	0.749 *	−0.221 *	0.484 *
	ΔB	−0.505 *	0.740 *	−0.306 *	0.537 *

## Data Availability

Data are contained within the article and Supplementary Materials.

## Supplementary Materials

The following supporting information can be downloaded at https://www.mdpi.com/article/10.3390/plants15081184/s1. Figure S1: Dynamics of ΔR, ΔG, and ΔB under three steps of light intensity (20 min for 76 μmol m^−2^ s^−1^, 20 min for 756 μmol m^−2^ s^−1^, and 10 min for 76 μmol m^−2^ s^−1^); Figure S2: Dynamics of ΔR, ΔG, and ΔB under three steps of light intensity (10 min for 76 μmol m^−2^ s^−1^, 20 min for 756 μmol m^−2^ s^−1^, and 20 min for 76 μmol m^−2^ s^−1^); Figure S3: The localization of devices and plants during experiments and example of ROI placements on plants and standards of reflectance; Figure S4: Relation of the measured values in red (R), green (G), and blue (B) spectral channels of RGB camera to similar values, which were calculated based on duration of exposure (i.e., to real light sums for these channels); Figure S5: Example of intensities of light reflected from the wheat leaf and from the white and gray reflectance standards, which were measured using the RGB camera; and Figure S6: The scheme of the minimal optical model based on the Kubelka–Munk theory and the scheme of simulation of shift in linear light absorption coefficient in leaf induced by illumination (i.e., the scheme of imitation of the heterogeneous distribution of chloroplasts in the leaf cells under illumination).
