# Supplementary material for: Light-Induced Changes in RGB Reflectance Parameters in Wheat and Pea Leaves in the Minute Range
Source: Plants (Basel). 2026 Apr 12;15(8):1184. doi: 10.3390/plants15081184 (PMC13119824; doi:10.3390/plants15081184)
Supplement: Supplementary file 1 [file plants-15-01184-s001.zip › plants-4207609-supplementary.pdf]

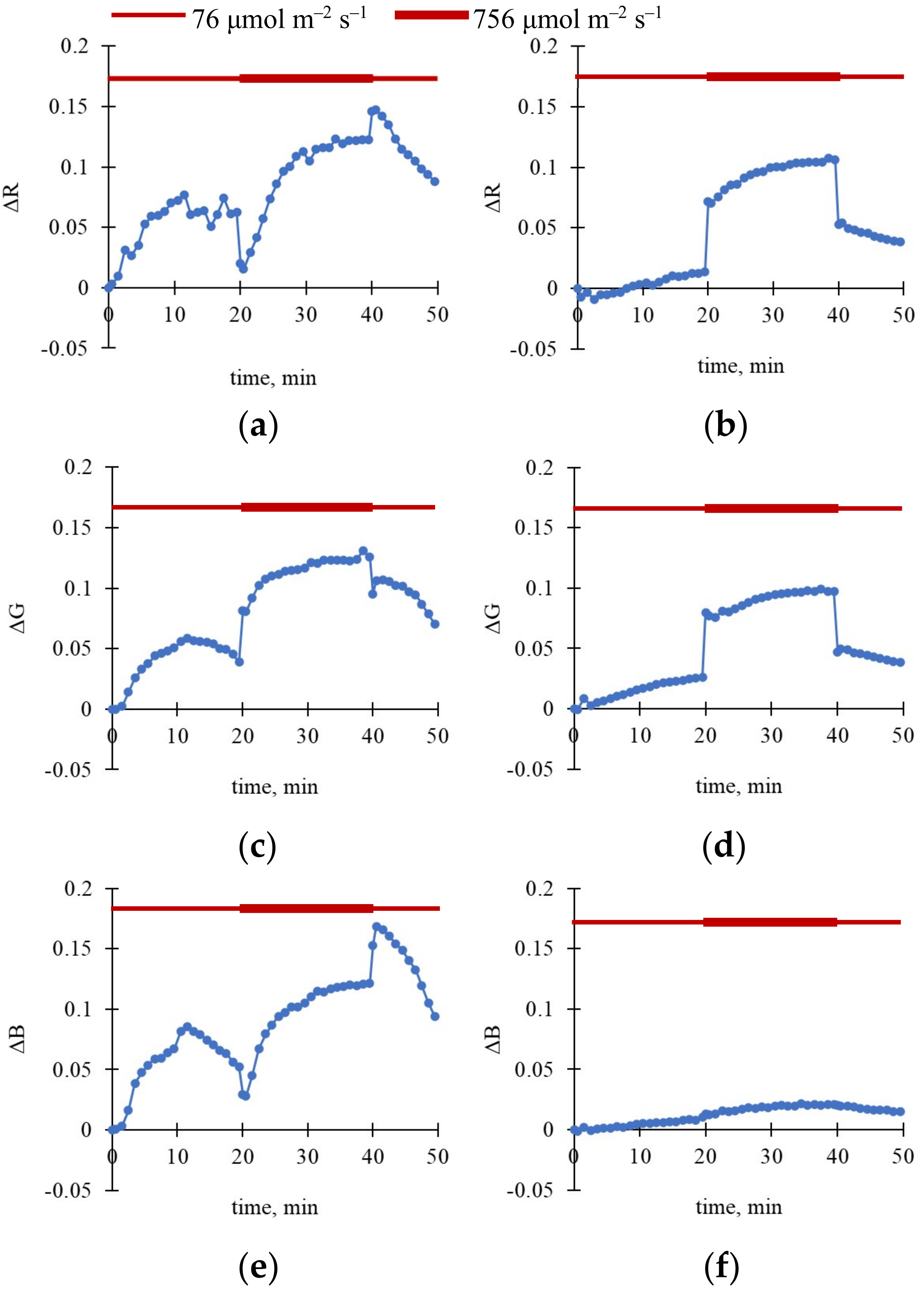

Figure S1. Typical dynamics of ΔR, ΔG, and ΔB under three steps of light intensity (20 min for 76 μmol m^{-2} s^{-1}, 20 min for 756 μmol m^{-2} s^{-1}, and 10 min for 76 μmol m^{-2} s^{-1}). ΔR in wheat (**a**) and pea (**b**), ΔG in wheat (**c**) and pea (**d**), and ΔB in wheat (**e**) and pea (**f**) are shown. ΔR, ΔG, and ΔB were calculated as differences between values, which were measured in the current time, and ones, which were measured immediately after turn on of light.

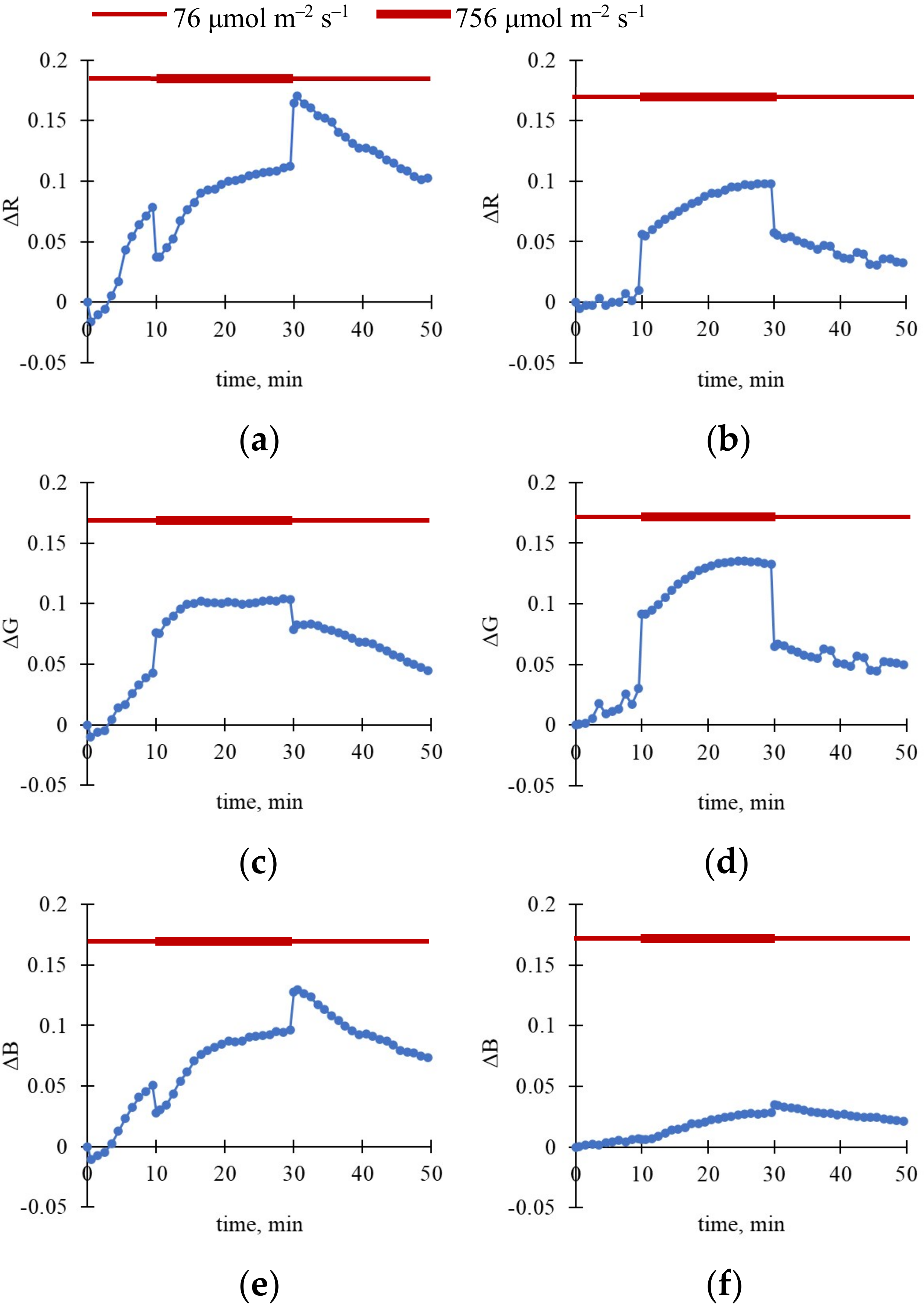

Figure S2. Typical dynamics of ΔR, ΔG, and ΔB under three steps of light intensity (10 min for 76 µmol m^{-2} s^{-1}, 20 min for 756 µmol m^{-2} s^{-1}, and 20 min for 76 µmol m^{-2} s^{-1}). ΔR in wheat (**a**) and pea (**b**), ΔG in wheat (**c**) and pea (**d**), and ΔB in wheat (**e**) and pea (**f**) are shown. ΔR, ΔG, and ΔB were calculated as differences between values, which were measured in the current time, and ones, which were measured immediately after turn on of light.

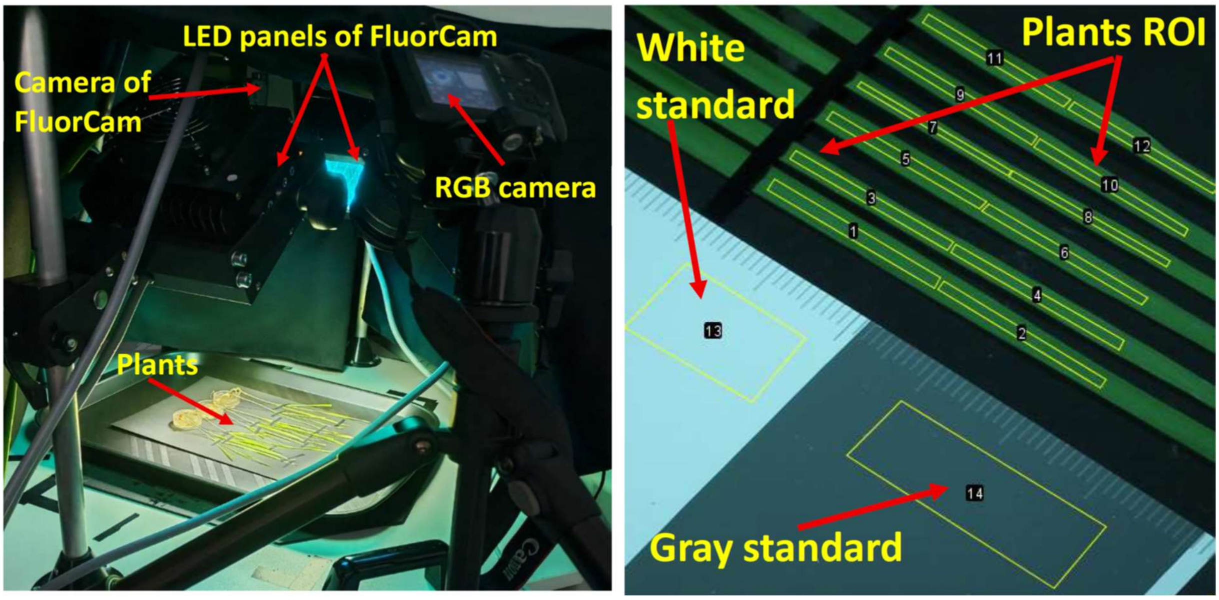

(a) (b)

Figure S3. The localization of devices and plants during experiments **(a)** and example of ROI placements on plants and standards of reflectance **(b)**.

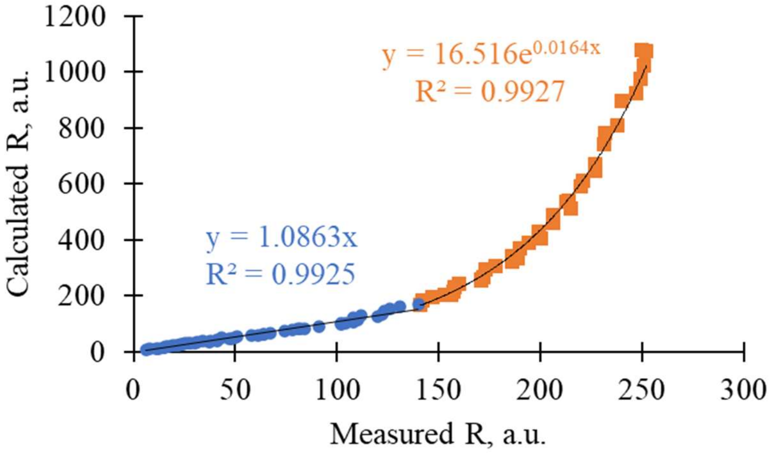

(**a**)

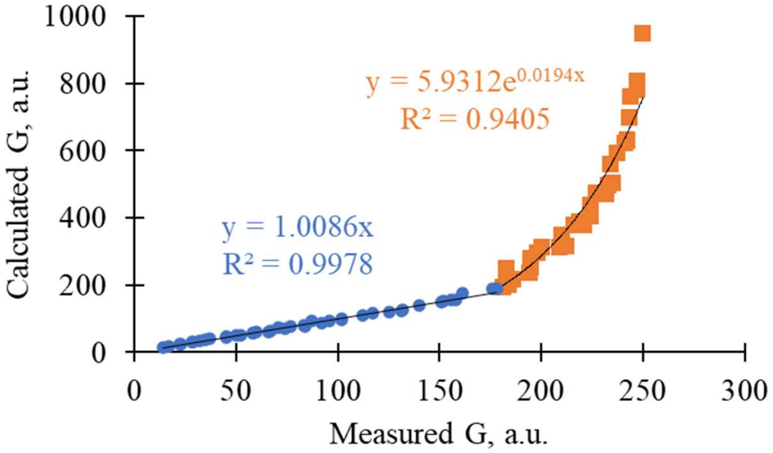

(**b**)

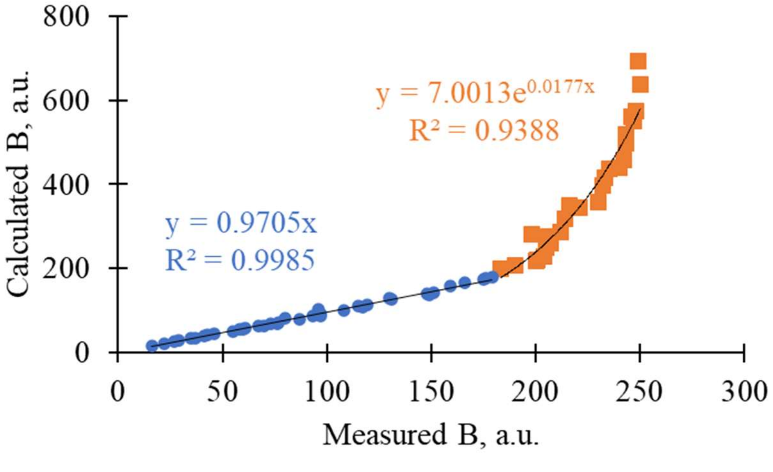

(**c**)

Figure S4. Relation of the measured values in red (R) **(a)**, green (G) **(b)**, and blue (B) **(c)** spectral channels of RGB camera to similar values, which were calculated based on duration of exposure (i.e., to real light sums for these channels). The white reflectance standard was used for measurements; Open FluorCam FC 800-O/1010-S was used as source of the white actinic light. The blue markers show points, which can be described by linear regression, the orange markers show points, which can be described by exponential regression. Arbitrary units, which were measured by cameras, were used for values in R, G, and B spectral channels. Values of these units ranged from 0 to 255 for measured values (values >250 arbitrary units were not analyzed to minimize errors) and from 0 to arbitrary for calculated values.

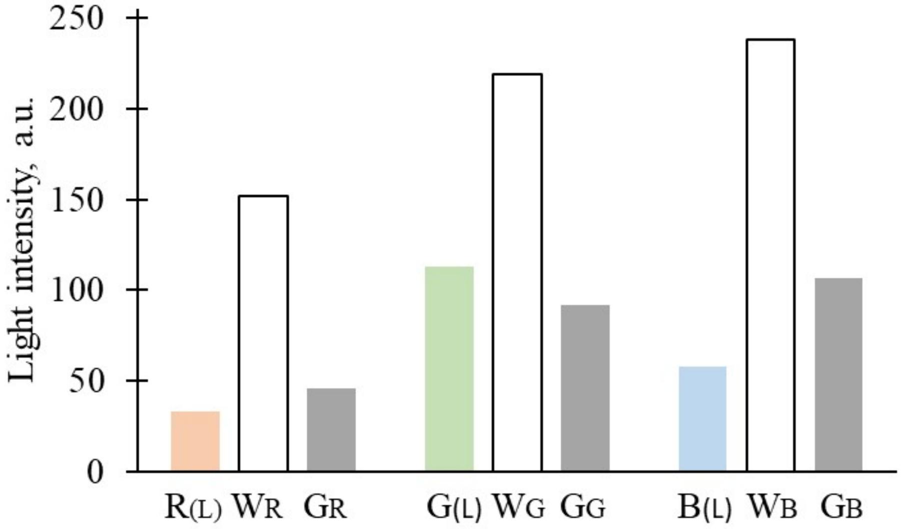

Figure S5. Example of intensities of light reflected from the wheat leaf and from the white and gray reflec-tance standards, which were measured using the RGB camera. R(L), G(L), and B(L) are intensities of red, green, and blue light reflected from wheat leaf; WR, WG, WB are intensities of red, green, and blue light reflected from the white standard; GR, GG, GB are intensities of red, green, and blue light reflected from the grey standard. Measurements of the parameters were simultaneous.

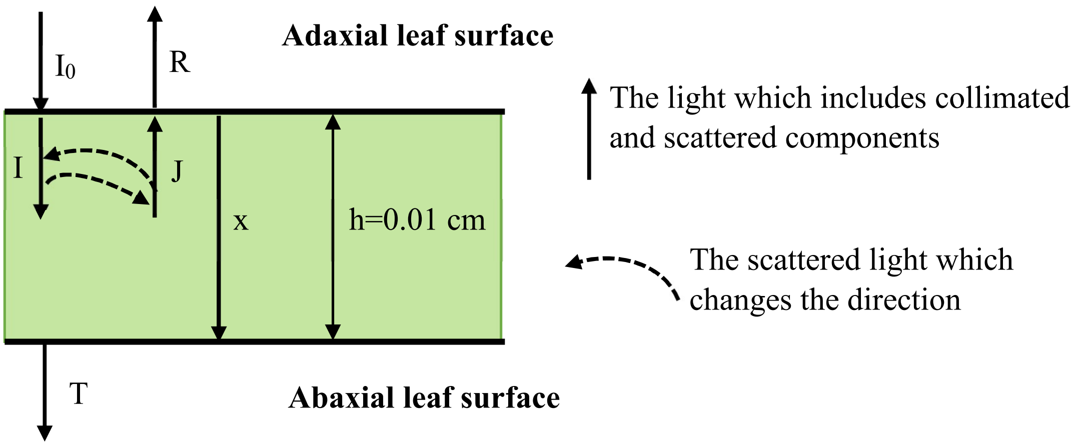

(a)

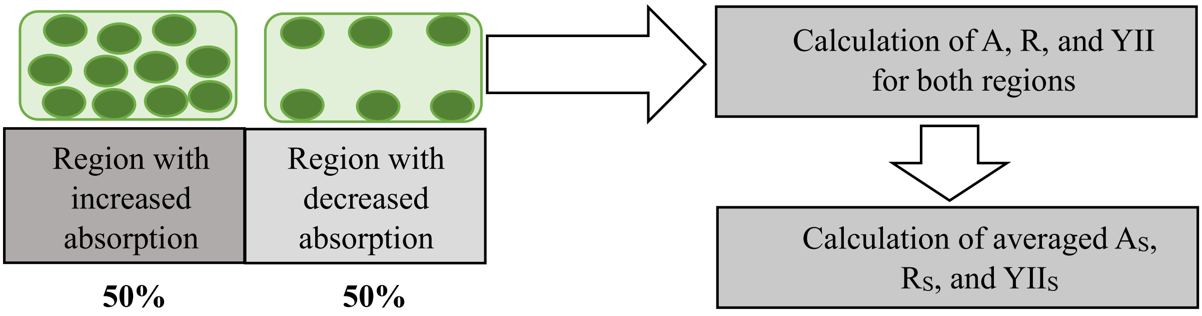

(b)

Figure S6. The scheme of the minimal optical model based on the Kubelka–Munk theory **(a)** and the scheme of simulation of shift in linear light absorption coefficient in leaf induced by illumination (i.e., the scheme of imitation of the heterogeneous distribution of chloroplasts in the leaf cells under illumination). **(b)**. I_0 is incident light from light source; I and J are is forward and backward light flows, respectively; R and T are reflectance and transmittance of the leaf, respectively; h is leaf thickness; x is coordinate axis. A, R, and YII are absorption, reflectance and the quantum yield of photosystem II; A_S, R_S, and YII_S are A, R, and YII, which were averaged for regions with increased and decreased the linear light absorption.
